# Multimodal treatment of aggressive forms of breast cancer

**Published:** 2014-09-25

**Authors:** D Mihai, S Voiculescu, D Cristian, F Constantinescu, E Popa, T Burcos

**Affiliations:** Surgery Clinic, “Coltea" Hospital, Bucharest

**Keywords:** invasive form, differentiation degree, receptors for estrogen, progesterone and HER2

## Abstract

Abstract

Aggressive breast cancer is an invasive form with G3, G4 differentiation degree, the absence of receptors for estrogen and progesterone and the absence or presence of HER2 (+ or 3+) gene. The final diagnosis is established by cumulating the clinical, paraclinical, histopathological and immunohistochemical diagnosis.

Material and method: 84 out of 268 aggressive breast cancer cases were presented in the study, which were operated in October 2011-September 2013. The inclusion and exclusion criteria are exposed in the study lot and the treatment schemes.

Results: For the study lot (lot A made up of 36 cases, lot B made up of 41 cases, lot C made up of 7 cases) the distribution was presented on age groups, histopathological and immunohistochemical classification, etiologic factors, type of surgery, postoperative staging and complications.

Conclusions: The treatment of aggressive breast cancer depends on the level of the aggressiveness of the disease, the biologic status and the age that imposes the order of chemotherapy, radiotherapy, surgical treatment and target therapy.

## Introduction

Aggressive breast cancer represents one third of the breast cancers, being diagnosed in young people but also in people aged 51-90, in the last couple of years. The most important factors involved in the appearance of the aggressive factors are the endogen and genetic ones. The most efficient therapeutic strategy for the aggressive breast cancer is established according to the stage, disease aggressiveness, age and biologic status of the patient. 

## Material and method 

For a period of 2 years, between October 2011 and September 2013, 268 patients suffering from breast cancer have been hospitalized and operated in “Coltea" Surgery Clinic, out of whom 84 patients - 31,34% (82 women and 2 men) have presented aggressive forms of cancer. The diagnosis of aggressive breast cancer has been established preoperatory by cumulating the clinical, paraclinical and certainty (histopathologic and immunohistochemical) diagnosis. 

 The inclusion criteria were the following: the breast cancers diagnosed by biopsy or tumor excision, which have presented clinical, biologic and histopathological aggressiveness characters. 

The exclusion criteria were the following: the breast cancers that have appeared during pregnancy or when there were other pathologies involved. 

 The 84 patients were split in 3 lots: 

 -lot A made up of 36 patients (35 women plus one man) did not receive a neoadjuvant treatment. 

 -lot B made up of 41 patients (40 women plus one man) have received a PCT neoadjuvant treatment. 

 -lot C made up of 7 patients, who have benefited from a PCT+RT or RT neoadjuvant treatment. 

 The groups’ distribution according to age for each lot is presented in Table 1.

**Table 1:** The stadialization according to age groups

Age groups	21-30	31-40	41-50	51-60	61-70	71-80	>81years old
LOT A		4	3	9	10	10	0
LOT B		3	3	19	11	5	0
LOT C	1	0	0	3	0	1	2
	1(1,19)	7(8,33)	6(7,14)	31(36,9)	21(25)	16(19,04)	2(2,38)

## Results 

Lots of parameters have been used in the study group, as it follows: 

 - From the histopathological point of view, the following forms of cancer have been diagnosed: CDI 43 cases =51,19%; CLI 7 cases = 8,33%; CDI+CLI 16 cases = 19,04%; methaplasic carcinoma 11 cases = 13,09% and CDI plus other forms 5 = 5,95%. 

 The histopathologic examination for each lot of patients is presented in Table 2.

**Table 2:** Immunohistochemical classification on lots of patients

HP Forms	CDI	CLI	mixed	metaplasic	CDI+other
LOT A	21	2	7	3	3
LOT B	19	4	8	7	3
LOT C	3	1	1	1	1

 - From the immunohistochemical point of view, the results were the following: 

 ER-PR-HER- 73 cases = 86,9% 

 ER-PR-HER+ 8 cases = 9,52% 

 ER-PR-HER+++ 3 cases = 3,57% 

 The immunohistochemical exam for each lot of patients is presented in Table 3. 

**Table 3:** Immunohistochemical classification

IHC Forms	ER-PGR-HER2-	ER-PGR-HER+	ER-PGR-HER+++
LOT A	34 (94,4%)	1 (2,77%)	1 (2,77%)
LOT B	33 (80,48%	6 (14,63%)	2 (4,87%)
LOT C	5 (71,42%)	2 (28,57%)	0

 from the etiological point of view, each of the 3 types of risk factors had an important role: 

 - environmental factors: breast trauma, stress and smoking in 5 cases = 9,09% 

 -endogenous factors: nulliparousness, absence of breastfeeding, breast benign tumors, endocrine disorders, obesity = 37 cases – 67,27%. 

 -genetic factors: BRCA1, BRCA2 genes mutations and familial aggregation syndrome 13 cases = 23,63%. 

 -from the symptoms point of view, all 84 patients presented to the physician according to the stage of the disease: pain, reaction of the tegument and axillary adenoplastic types. 

 -the pTNM postoperative staging has been the following: stage I – 7 cases (8,33%), stage II – 35 cases (41,66%), stage III – 15 cases (17,84%) and stage IV – 27 cases (32,14%). 

 The staging for each lot of patients is presented in Table 4. 

**Table 4:** Postoperative staging

pTNM	stage I	stage II	stage III	stage IV
LOT A	4	17	4	11
LOT B	3	15	9	14
LOT C	0	3	2	2

 -the surgeries undergone were the following: 

 -radical mastectomy Madden type – 72 cases (85,71%) 

 -simple mastectomy – 2 cases (2,38%) 

 -simple mastectomy + axillary lymphadenectomy – 1 case (1,19%) 

 -qadrantectomy + axillary lymphadenectomy – 7 cases (8,33%) 

 -simple mastectomy + partial resection of the breast muscles – 1 case (1,19%) 

 -Madden mastectomy + partial resection of the invaded breast muscles – 1 case (1,19%) 

 The surgeries undergone for each of the 3 lots of patients are presented in Table 5. 

**Table 5:** 

SURGERIES	LOT A	LOT B	LOT C
radical mastectomy Madden type	28	37	7
simple mastectomy	2		
simple mastectomy + axillary ly	1		
simple mastectomy + partial resection of the muscles		1	
Madden mastectomy + partial resection of the muscles		1	
quadrantectomy + axillary ly	5	2	

 The main therapy is the surgical one, and together with PCT, RT, aimed modern therapy have earned ground, being able to be undergone as a neoadjuvant or adjuvant treatment. 

 - postoperatively, the evolution has been favorable, the main complication being the lymphology determined by the presence of axillary adenopathy; this being present in 48 cases as it follows: 

 -stage I 4 cases (8,33%) 

 -stage II 11 cases (22,91%) 

 -stage III 12 cases (25%) 

 -stage IV 21 cases (43,75%) 

 - The mortality and morbidity rate has been 0. 

## Discussions

Aggressive breast cancer is an invasive form with G3, G4 differentiation degree, absence of receptors for estrogen and progesterone and presence of HER gene (+ or 3+) or its absence. 

 The factors involved in the appearance of breast cancer are classified as it follows: 

 -environment factors – irradiation of thoracic area, a diet rich in proteins, fats and light sweets, exposure of the breast to ultraviolet radiations, mammary traumatisms, alcohol, smoking, stress. 

 The traumatisms (two cases) and smoking (three cases) had an important role in the appearance of breast cancer in some patients in lot A. 

 -endogenous factors – precocious menarche age, late menopause, nulliparity, absence of breast feeding, obesity, precancerous states, benign tumors of the mammary gland, immune deficits, endocrine disorders. 

 The nulliparity (four cases in lot A + two cases in lot B), benign tumors (two cases in lot A + one case in lot B), obesity (nine cases in lot A + six cases in lot B + one case in lot C), endocrine disorders (one case in lot A + four cases in lot B), absence of breast feeding (four cases in lot A + three cases in lot B), were the most important endogen factors. 

 -genetic factors: 

 a) direct heritage of the specific genetic defects, such as BRCA1 gene mutations, which take place at the level of chromosome 1 [**10**]. 

 b) modified transmission of BRCA2 gene leads to the appearance of breast cancer and especially to the appearance of ovarian cancer [**10**]. 

 Lynch [**1**] described the presence of many colonic, gastric and breast cancers to the members of the same family. 

 The role of the genetic factors in the appearance of breast cancer in the three lots of study is presented as it follows: 

 Lot A – breast neoplasm (four cases), gastric neoplasm (one case), colon neoplasm (one case), genital neoplasm (one case). 

 Lot B – breast neoplasm (two cases), colon neoplasm (one case), gastric neoplasm (one case). 

 Lot C – breast neoplasm (one case), gastric neoplasm (one case). 

 The clinical, paraclinical and certainty diagnoses together establish a positive diagnosis of aggressive breast cancer. 

 The clinical diagnosis highlights through the inspection and palpation of both breasts, the supraclavicular fossae and the axillary region, the location of the tumor, the presence of the axillary adenopathy and the nipple discharges. 

 The paraclinical diagnosis is established through a digital mammography [**2**] or a stereotaxic mammography, breast ultrasound [**3**], tridimensional ultrasound [**4**], Doppler ultrasound, MRI and elastography. 

 In establishing the paraclinical diagnosis for the three lots of patients, the following investigations have been made: mammography, classical ultrasound, elastography and mammary MRI. 

 - Lot A:mammography + ultrasound (twenty five cases) 

 o mammography + elastography (eleven cases) 

 - Lot B: mammography (twenty cases). 

 mammography + elastography (fifteen cases). 

 ultrasound + MRI (six cases). 

 - Lot C: mammography + ultrasound (two cases). 

 ultrasound + elastography (two cases). 

 ultrasound + breast MRI (three cases). 

 The laboratory diagnosis is sustained at the level of antigens CA-15-3, TAG 72, MCA and D cathepsins. 

 The level of the tumoral markers for the three lots of patients was the following: 

 - Lot A: four cases stage I, eight cases stage II, four cases stage III, ten cases stage IV. 

 - Lot B: one case stage I, ten cases stage II, seven cases stage III, fourteen cases stage IV. 

 - Lot C: two cases stage II, two cases stage III, two cases stage IV. 

 The certainty diagnosis is obtained by cumulating the histopathological and immunohistochemical diagnoses. 

 The histopathological diagnosis is established by a percutaneous biopsy of the breast, imagistically guided through the vacuum-assisted biopsy or though a sectoral mammary resection with a HP exam, this way determining the form and the degree of histopathological differentiation [**5**]. The IHC is determined and classified also by taking into account the histologic type of the tumor. This way, the classification from the histopathologic point of view of the aggressive forms of breast cancer is ductal, lobular, mixed, metaplastic and tubular [**6**]. 

 From the immunohistochemical point of view, the aggressive forms can be classified as it follows: ER-, PGR, HER-, ER-PGR-HER + and ER-PGR-HER+++ [**7**]. 

 The result of the immunohistochemical tests for the three lots of patients is the following: 

 - Lot A: four cases ER-, PGR-, HER-stage I 

 Fifteen cases ER-, PGR-, HER -; one case ER-PGR-HER+; one case ER-PGR-HER+++ - stage II. 

 Four cases ER-, PGR-, HER- , stage III. 

 Eleven cases ER-, PGR-, HER -, stage IV. 

 - Lot B: three cases ER-, PGR-, HER-, stage I. 

 Ten cases ER-, PGR-, HER-, four cases ER-PGR-HER+, one case ER-PGR-HER+++ - stage II. 

 Eight cases ER-, PGR-, HER-, one case ER-PGR-HER+++ - stage III. 

 Twelve cases ER-, PGR-, HER-, two cases ER-PGR-HER+, stage IV. 

 - Lot C: two cases ER-PGR-HER-, one case ER-PGR-HER+, - stage II. 

 One case ER-PGR-HER-, one case ER-PGR-HER+, stage III. 

 Two cases ER-PGR-HER-, stage IV. 

 For the staging of the aggressive forms, the following investigations can also be used: breast MRI, bone CT, CT/ MRI/ abdominal and pelvic CT, pulmonary Rx and scintigraphy. 

 The individual therapy of breast cancer is correspondent to the stage of the disease, the histopathological form and the immunohistochemical diagnosis, which impose the order of the surgical neoadjuvant and adjuvant treatment [**8**]. 

 There are 3 schemes of treatment which are used: 

 1. surgical treatment followed by an adjuvant treatment; 

 2. neoadjuvant treatment (PCT) plus surgical treatment + adjuvant treatment after the oncologic reevaluation; 

 3. neoadjuvant treatment (PCT+RT or RT) + plus surgical treatment + adjuvant treatment after the oncologic reevaluation. 

 Types of mastectomy 

 Madden-the two pectoral muscles are preserved. 

 Patey-the breast and the anterior fascia of the high chest muscle are lifted, the axillary lymphoganglionar tissue suppressing the low chest muscle. 

 Modified Halsted–the breast, the chest muscles and the axillary lymphoganglionar tissue are removed in bloc when the local situation allows it. 

 The conservative treatment – wide sectorectomy and quadrantectomy associated or cumulated with the axillary dissection according to the ganglionar stages. 

 *The main treatment for stage I and II is surgically preceded or not by the neoadjuvant treatment and followed by an adjuvant treatment. 

 The surgical treatment can be conservatory (quadrantectomy associated with axillary lymphadenectomy) or radical (mastectomy followed or not by a breast reconstruction), followed in both of the cases by an adjuvant treatment (PCT + or – HER 2 +RT anti antibodies. 

 The following types of surgeries were done in the study group, for the three lots of patients: 

 - Lot A: stage I: two modified Madden mastectomies; two quadrantectomies + axillary lymphadenectomy. 

 stage II: fourteen Madden mastectomies; three quadrantectomies + axillary lymphadenectomy. 

 - Lot B: stage I: three modified Madden mastectomies; 

 stage II: fourteen Madden mastectomies; one quadrantectomy + axillary lymphadenectomy. 

 - Lot C: stage II: three modified Madden mastectomies. 

 *For the IIIrd and IVth stages, the surgical treatment is preceded by PCT + or HER 2 + RT anti antibodies neoadjuvant treatment. 

 The surgical treatment can be conservatory, followed by adjuvant treatment (breast RT, infraclavicular, supraclavicular and internal breast chain + PCT + or- HER 2 anti antibodies. 

 Radically, when followed by an adjuvant treatment (thoracic, infraclavicular, supraclavicular, internal breast chain + PCT + or – HER 2 anti antibodies RT); breast reconstruction can be done after radiotherapy is ended. 

 In case there is a negative answer after the neoadjuvant treatment, PCT is continued while associated with RT; in case the answer is positive, surgical and adjuvant treatment is applied according to the previous scheme; in the cases in which the answer is negative individual treatment is applied. 

 The type of surgical interventions undergone for the three lots of patients is the following: 

 - Lot A: stage III four modified Madden mastectomies. 

 Stage IV eight modified Madden mastectomies: two simple mastectomies; one simple mastectomy with axillary lymphadenectomy. 

 - Lot B: stage III nine modified Madden mastectomies. 

 Stage IV eleven modified Madden mastectomies; one modified Madden mastectomy + partial resection of the chest muscles; one simple mastectomy + a partial resection of the chest muscles; one quadrantectomy + one axillary lymphadenectomy. 

 - Lot C: stage III two modified Madden mastectomies. 

 Stage IV two modified Madden mastectomies. 

 The palliative treatment is applied in the following cases: brain, pleural, pericardial, bone and thoracic metastases or biliary and urethral obstruction, fracture impediment or pathological fracture. The treatment approached is surgical associated with chemotherapy and radiotherapy. 

 The postoperative complications are the following: 

 -subcutaneous (ejection) hematoma; lymph drains from the dissection location (drainage and repeated ejections); axillary suppuration of the wound; necrosis of the cutaneous edges or of some skin areas (may need the excision of some suture wires or their correct delimitation, excision and secondary suture); thrombophlebitis of the leg (anticoagulant treatment); pulmonary or pleural complications. 

 The only complication in the study group was lymphology; the distribution according to the three lots of patients is the following: 

 - Lot A: two cases – stage I, five cases – stage II, three cases – stage III, nice cases – stage IV. 

 - Lot B: two cases – stage I, four cases – stage II, seven cases – stage III, eleven cases – stage IV. 

 - Lot C: two cases – stage II, two cases – stage III, two cases – stage IV. 

 The postoperative sequelae are the following: 

 -the functional difficulty of the arm can appear after the damaging of the nerve of the serratus anterior muscle, which determines the anterior movement of the scapula. 

 -the axillary cutaneous clip or the scar, located in a delicate flexion area, can entail correction surgeries.

 -the short skin syndrome may determine the most important functional difficulty. 

 -episodically or permanently, the soft or hard arm edema can be considered the result of the lymphatic barrage of some sepsis elements or of an aseptic sclerosis or local relapses. 

 The long-distance lymphatic and blood metastases are located in the lungs, bones, liver, suprarenal glands, ovaries, teguments, CNS. 

 A particular metastasis form is represented by pleurisy, ascites and pulmonary lymphangitis. 

 The therapeutic conduct applied in the case of a local or regional relapse, after the conservative treatment is the following: 

 -breast relapse (the positive diagnosis is based on the clinical examination and breast MRI and presupposes a total mastectomy + or – RT. 

 -the axillary relapse imposes resection and radiotherapy at the level of the thorax, of the supra and infraclavicular fossae and the axillary area. 

 -the supraclavicular relapse imposes radiotherapy at the level of the thorax, of the supra and infraclavicular fossae. 

 -the relapse at the level of the internal ganglionar breast chain imposes radiotherapy at the level of the thorax, of the supra and infraclavicular fossae and the internal breast chain. 

 The local relapse after mastectomy imposes radiotherapy at the level of the thorax. 

 The moment of the surgery, the strategy and the surgery technique have been adapted to each case, taking into account the following parameters: 

 -the general state of the patient at admission; 

 -the existence of other organic flows (HTA, IC, DZ, etc.) 

 -the necessity of doing the surgery in emergency conditions (hemorrhage, infection, tumor necrosis) 

 -the postoperative result is characterized by the following elements: 

 1. clinical examination (inspection, breasts palpation and genital examination) 

 2. mammography, ultrasound, MRI and elastography, puncture, biopsy. 

 3. abdomen and pelvis ultrasound (CT or MRI) 

 4. pulmonary Rx. 

 5. other investigations according to the organic flows. 

 The prognosis of the aggressive breast cancer depends on demographic characteristics (age, menopause status), tumors (lymphatic ganglionar status, tumor dimension, tumor size, pathogenic type) and biological markers (presence or absence of HER2).

## Conclusion 

The treatment of aggressive breast cancer depends on the stage of the aggressiveness of the disease, the biologic status and the age of the patient. The treatment presupposes a radical mastectomy and is cumulated with the neoadjuvant and adjuvant treatment (represented by PCT, RT, targeted modern therapy – antiHER2 monoclonal antibodies). 

 The interdisciplinary collaboration between the surgeon, oncologist, radiotherapist and family doctor is very important. Moreover, the patient-physician communication must be based on correct, complete and updated information, regarding the essential therapeutic possibilities. 
